# Perceived Economic Burden, Mortality, and Health Status in Patients With Heart Failure

**DOI:** 10.1001/jamanetworkopen.2024.1420

**Published:** 2024-03-21

**Authors:** Yuan Yu, Jiamin Liu, Lihua Zhang, Runqing Ji, Xiaoming Su, Zhiping Gao, Shuang Xia, Jing Li, Liwen Li

**Affiliations:** 1 Department of Cardiology, Guangdong Cardiovascular Institute, Guangdong Provincial People’s Hospital, Guangdong Academy of Medical Sciences, Guangzhou, China; 2 National Clinical Research Center of Cardiovascular Diseases, National Health Commission Key Laboratory of Clinical Research for Cardiovascular Medications, State Key Laboratory of Cardiovascular Disease, Fuwai Hospital, National Center for Cardiovascular Diseases, Chinese Academy of Medical Sciences and Peking Union Medical College, Beijing, China; 3 Department of General Practice, Guangdong Provincial People’s Hospital, Guangdong Academy of Medical Sciences, Guangzhou, China

## Abstract

**Importance:**

In the face of an emerging heart failure (HF) epidemic, describing the association between perceived economic burden (PEB) and health care outcomes is an important step toward more equitable and achievable care.

**Objectives:**

To examine the association between PEB and risk of 1-year clinical outcomes and HF–specific health status in patients with acute decompensated HF.

**Design, Setting, and Participants:**

This prospective, multicenter, hospital-based cohort study prospectively enrolled adult patients hospitalized for acute decompensated HF at 52 hospitals in China from August 2016 to May 2018, with 1-year follow-up. Data were analyzed on June 17, 2022.

**Exposure:**

Perceived economic burden, categorized as severe (cannot undertake expenses), moderate (can almost undertake expenses), or little (can easily undertake expenses).

**Main Outcomes and Measures:**

The clinical outcomes of the study were 1-year all-cause death and rehospitalization for HF. Heart failure–specific health status was assessed by the 12-Item Kansas City Cardiomyopathy Questionnaire (KCCQ-12).

**Results:**

Among 3386 patients, median age was 67 years (IQR, 58-75 years) and 2116 (62.5%) were men. Of these patients, 404 (11.9%) had severe PEB; 2021 (59.7%), moderate PEB; and 961 (28.4%), little PEB. Compared with patients with little PEB, those with severe PEB had increased risk of 1-year mortality (hazard ratio [HR], 1.61; 95% CI, 1.21-2.13; *P* < .001) but not 1-year HF rehospitalization (HR, 1.21; 95% CI, 0.98-1.49; *P* = .07). The mean (SD) adjusted KCCQ-12 score was lowest in patients with severe PEB and highest in patients with little PEB at baseline (40.0 [1.7] and 50.2 [1.0] points, respectively; *P* < .001) and at each visit (eg, 12 months: 61.5 [1.6] and 75.5 [0.9] points respectively; *P* < .001). Patients reporting severe PEB had a clinically significant lower 1-year KCCQ-12 score compared with those reporting little PEB (mean difference, −11.3 points; 95% CI, −14.9 to −7.6 points; *P* < .001).

**Conclusions and Relevance:**

In this cohort study of patients with acute decompensated HF, greater PEB was associated with higher risk of mortality and poorer health status but not with risk of HF rehospitalization. The findings suggest that PEB may serve as a convenient tool for risk estimation and as a potential target for quality-improvement interventions for patients with HF.

## Key Points

**Question:**

Is perceived economic burden an independent factor associated with prognosis for patients with heart failure?

**Findings:**

In a large national cohort study of 3386 patients hospitalized for acute decompensated heart failure in China, greater perceived economic burden was associated with higher risk of 1-year all-cause mortality and worse health status but not with rehospitalization for heart failure.

**Meaning:**

Perceived economic burden may serve as a convenient tool for estimating the risk of poor health outcomes and as a potential target for quality-improvement interventions.

## Teaser

This cohort study investigates whether perceived economic burden is associated with all-cause mortality and rehospitalization for heart failure among patients with acute decompensated heart failure.

## Introduction

Heart failure (HF) affects about 13.7 million patients in China,^1^ and an estimated 3.0 million Chinese adults have incident HF each year.^2^ Patients with HF face the challenge of high medical costs due to recurrent hospitalization and long-term therapy. Difficulties in affording health care have raised widespread public concern in both the US^3,4,5,6,7^ and China.^8,9^ The economic burden perceived by patients is a composite perception of factors, including income, living expenses, out-of-pocket payment, and individual satisfaction, and is regarded as a more thorough assessment of financial challenges than subjective measurement.^6^ Describing the association between perceived economic burden (PEB) and outcomes is an important step toward more equitable and achievable care.

Several prior studies have focused on the potential adverse consequences of PEB for patient-reported outcomes. They demonstrated that difficulty in affording medical care was associated with impaired quality of life, well-being, and mental health.^10,11,12^ Nevertheless, these studies were limited by their small size. Additionally, a prior study found that financial barriers to health care services and medications were associated with increased rates of rehospitalization but not mortality after acute myocardial infarction.^13^ It remains unknown whether patients’ perceived difficulties in affording medical care are associated with clinical events among patients with HF. To address these knowledge gaps, we assessed the PEB in a nationwide prospective cohort of patients hospitalized for acute decompensated HF (ADHF) in China and examined the association of PEB with clinical outcomes (death and rehospitalization) and HF-specific health status within 1 year of discharge.

## Methods

### Study Design and Population

This cohort study followed the Strengthening the Reporting of Observational Studies in Epidemiology (STROBE) reporting guideline. This analysis was based on the China Patient-Centered Evaluative Assessment of Cardiac Events Prospective Heart Failure (PEACE 5p-HF) study, a prospective multicenter cohort study.^14^ The China PEACE 5p-HF study consecutively screened local patients hospitalized for HF within 48 hours of admission between August 2016 and May 2018 at 52 diverse hospitals (48 tertiary and 4 secondary hospitals) located in 20 provinces covering all economic and geographic regions in China. The eligibility criteria included age 18 years or older and hospitalization for HF (hereafter, HF hospitalization). Patients who signed informed consent were enrolled in the prospective cohort. Enrolled patients completed a baseline interview during the index hospitalization and were followed up at 1, 6, and 12 months after discharge. If patients were unwilling or unable to attend their scheduled in-person follow-up visits, information was obtained by telephone through direct correspondence with patients, their relatives, or their physicians. For this analysis, we included the patients enrolled in the China PEACE 5p-HF study who were admitted for ADHF, answered the questions about PEB during the index hospitalization, and were discharged alive. The ethics committee of Fuwai Hospital and the local ethics committees at the included sites approved this study.

### Data Collection

Patient demographics, socioeconomic status (ie, marital status, educational level, family annual income, and medical insurance), smoking status, PEB, and self-reported health status were collected by physicians using a standardized questionnaire through a face-to-face interview during the index hospitalization. Left ventricular ejection fraction (LVEF) was measured during the index hospitalization by trained physicians using a standard protocol. Information about patients’ comorbidities, clinical characteristics at discharge (including heart rate and blood pressure), New York Heart Association (NYHA) functional class, local laboratory tests, treatments, and medical expenses was obtained from medical records from the index hospitalization.

Heart failure was categorized by LVEF: HF with reduced LVEF (<40%), HF with midrange LVEF (≥40% to <50%), and HF with preserved LVEF (≥50%). Comorbidities were obtained from discharge diagnosis or positive laboratory test results. For example, anemia was defined as a diagnosis of anemia or a hemoglobin level less than 12.0 g/dL in men or less than 11.0 g/dL in women (to convert to g/L, multiply by 10.0). Reduced kidney function was defined as an estimated glomerular filtration rate less than 60 mL/min/1.73 m^2^, calculated by an equation developed by adaptation of the Modification of Diet in Renal Disease equation based on data from Chinese patients with chronic kidney disease.^15^

The basic health insurance system in China consists of 3 schemes. Specifically, the New Cooperative Medical Scheme (NCMS) covers residents in rural areas, Urban Employee-Based Medical Insurance (UEBMI) covers urban residents who are employed, and Urban Resident-Based Medical Insurance (URBMI) covers urban residents not covered by the UEBMI scheme, including those who are unemployed, older individuals, and children. Medical expense of the index hospitalization indicated the sum of medical care during hospitalization. The annual out-of-pocket payment represented costs of all health care services after reimbursement in the past year.

We used the short version of the 12-item Kansas City Cardiomyopathy Questionnaire (KCCQ-12)^16^ to assess HF-specific symptoms, functioning, and quality of life in all enrolled patients. The scores range from 0 to 100, and a higher score reflects possibly better health-related quality of life. For KCCQ-12 summary scores of individual patients, a difference of approximately 3 to 5 points supports a minimal clinically important disparity in HF status and a difference of 10 to 17 points indicates moderate disparity.^16,17^ The questionnaire had been linguistically and culturally translated into simplified Chinese to ensure data validity.

### PEB Collection and Definition

We used an approach to evaluate patients’ PEB in health care that was similar to that used in a previous study.^11,18^ During the index hospitalization, patients were asked, “What do you think of the burden of medical expense in the past year?” Responses were “cannot undertake,” “almost undertake,” and “can undertake easily,” which were classified as severe, moderate, and little PEB, respectively. To validate the PEB evaluation, we asked another 2 questions related to severe economic burden: “During the past 12 months, have you ever borrowed money from others to pay for medical expense?” and “During the past 12 months, have you ever avoided health care due to costs?” We reported the proportions of answers to these 2 questions by the categories of PEB.

### Outcomes

The clinical outcomes of the study were 1-year all-cause death and HF rehospitalization. All events were further confirmed according to the national database and centrally adjudicated by trained clinicians according to standard criteria. Patients’ health status was measured by the KCCQ-12 within 48 hours after admission during the index hospitalization and at 1, 6, and 12 months after discharge.

### Statistical Analysis

Data were analyzed on June 17, 2022. Patients were categorized into 3 groups according to their PEB: little, moderate, and severe. Continuous variables were expressed as medians and IQRs and compared by the Jonckheere-Terpstra test.^19^ Categorical variables were summarized by frequencies with percentages and compared by the Cochran-Armitage trend test for binary variables and the Mantel-Haenszel trend test for ordered multiclass variables.

Kaplan-Meier survival analysis was used to compare cumulative risk of death and HF rehospitalization by PEB groups. The index time was discharge date of index hospitalization. Crude and multivariable-adjusted Cox proportional hazards regression models were performed to assess the association of PEB with respective outcomes. We reported estimated hazard ratios (HRs) and 95% CIs. We adjusted for potential confounding variables based on previous literature^20^ and clinical knowledge, including age, sex, socioeconomic status (marriage, income, educational level, and medical insurance), current smoking status, comorbidities (coronary artery disease, hypertension, nonischemic cardiomyopathy, atrial fibrillation, valvular heart disease, reduced kidney function, anemia, stroke, chronic obstructive pulmonary disease, peripheral artery disease, and diabetes), laboratory testing (serum sodium and N-terminal pro-B-type natriuretic peptide [NT-proBNP] levels), LVEF, NYHA functional class, systolic blood pressure at discharge, heart rate at discharge, and discharge medications (angiotensin-converting enzyme inhibitor [ACEI] or angiotensin receptor blocker [ARB], β-blocker, and aldosterone antagonist). Subgroup analyses were conducted to explore interactions with outcomes. Multivariable Cox proportional hazards regression analyses were repeated after stratifying patients into different subgroups as follows: (1) age 65 years or younger or older than 65 years; (2) men or women; (3) annual family income less than 30 000 renminbi (RMB) (<US $4516), 30 000 to 70 000 RMB (US $4516-$10 538), more than 70 000 RMB (>US $10 538), or unknown; and (4) medical insurance coverage by UEBMI, URBMI, NCMS, or other insurance.

When we did analyses of KCCQ-12 scores, we excluded patients who died within 1 year or missed KCCQ-12 measurement at any time point. We reported mean (SD) KCCQ-12 scores at baseline and each study visit occurring at months 1, 6, and 12. Scores were compared by the Mann-Kendall trend test among the 3 economic burden groups. To calculate the adjusted KCCQ-12 scores at each time point, a general linear model was used. Covariates were chosen based on previous literature^20^ and clinical judgment and included baseline age, sex, socioeconomic status (marriage status, income, educational level, and medical insurance), current smoking status, comorbidities (coronary artery disease, hypertension, nonischemic cardiomyopathy, atrial fibrillation, valvular heart disease, reduced kidney function, anemia, stroke, chronic obstructive pulmonary disease, peripheral artery disease, and diabetes), laboratory testing (serum sodium and NT-proBNP levels), LVEF, systolic blood pressure at discharge, heart rate at discharge, and discharge medications (ACEI or ARB, β-blocker, and aldosterone antagonist). Considering that the KCCQ-12 clinical summary score reflects the key concept of NYHA functional class (homogeneity)^21,22^ and the known limitations in interrater reproducibility,^23^ we did not adjust for NYHA functional class. To further assess the mean difference of 1-year health status among PEB groups, we used a generalized linear regression model and adjusted for the baseline KCCQ-12 score and aforementioned covariates. We reported changes in the KCCQ-12 score with 95% CIs.

All models were adjusted for patient characteristics and a random effect at the institutional level to account for the patients’ clustering within hospitals. In sensitivity analyses, we included baseline KCCQ-12 score as a covariate in the Cox proportional hazards regression models. We also explored the interactions of KCCQ-12 scores and PEB with clinical outcomes. We conducted a sensitivity analysis with only patients admitted with new-onset HF to explore the association between PEB and risk of 1-year death and HF hospitalization among them.

We created a dummy variable as unknown annual family income for patients who declined to answer this question. The rates of missing values for other variables ranged from 0.1% (heart rate) to 5.5% (LVEF). We used multiple imputation by the Markov chain Monte Carlo method based on various demographic and clinical variables. All statistical analyses were conducted with SAS, version 9.4 (SAS Institute Inc). The survival plot was made with GraphPad Prism, version 9.5 (GraphPad Software). All comparisons were 2-sided, and *P* < .05 was considered statistically significant.

## Results

### Baseline Characteristics

After excluding 55 patients who died during the index hospitalization, 944 patients who were admitted for new-onset HF, and 522 patients who refused to answer PEB questions, we included 3386 patients in this analysis. Median age was 67 years (IQR, 58-75 years); 2116 (62.5%) were men, and 1270 (37.5%) were women. A total of 1411 patients (41.7%) with HF had reduced LVEF; 818 (24.2%), midrange LVEF; and 1157 (32.4%), preserved LVEF. A total of 1474 patients (43.5%) had NYHA functional class IV. During the 12 months before index hospitalization, 407 patients (12.0%) had borrowed money from others to pay for medical expenses and 740 (21.8%) had declined health care due to concern about costs. A total of 404 patients (11.9%) had severe PEB; 2021 (59.7%), moderate PEB; and 961 (28.4%), little PEB. Patients with severe PEB were more likely to be younger, women, unmarried, and covered by NCMS and to have an NYHA class of IV, lower educational level, higher annual out-of-pocket medical expenses, and higher NT-proBNP level; they were less likely to be discharged with an ACEI or ARB or a β-blocker (Table). As severity of PEB increased, the likelihood of borrowing money from others to pay for medical expenses and of declining health care due to costs also increased (eTable 1 in Supplement 1).

**Table.** Baseline Characteristics of Included Patients by PEB

Characteristic	Patients^a^				*P* value for trend
	All (N = 3386)	Severe PEB (n = 404)	Moderate PEB (n = 2021)	Little PEB (n = 961)	
Age, median (IQR), y	67 (58-75)	63 (53-70)	67 (59-76)	67 (58-76)	<.001
Sex					
Men	2116 (62.5)	236 (58.4)	1200 (59.4)	680 (70.8)	<.001
Women	1270 (37.5)	168 (41.6)	821 (40.6)	281 (29.2)	
Current smoker	551 (16.3)	58 (14.4)	299 (14.8)	194 (20.2)	<.001
Married	2741 (81.0)	300 (74.3)	1641 (81.2)	800 (83.2)	<.001
High school education or above	955 (28.2)	66 (16.3)	485 (24.0)	404 (42.0)	<.001
Annual family income, RMB (US $)^b^					
<30 000 (<4516)	1185 (35.0)	270 (66.8)	714 (35.3)	201 (20.9)	.059
30 000-70 000 (4516-10 538)	1133 (33.5)	77 (19.1)	695 (34.4)	361 (37.6)	
>70 000 (>10 538)	449 (13.3)	10 (2.5)	193 (9.5)	246 (25.6)	
Unknown	619 (18.3)	47 (11.6)	419 (20.7)	153 (15.9)	
Medical insurance					
UEBMI	1445 (42.7)	83 (20.5)	820 (40.6)	542 (56.4)	.002
URBMI	619 (18.3)	85 (21.0)	378 (18.7)	156 (16.2)	
NCMS	1020 (30.1)	197 (48.8)	637 (31.5)	186 (19.4)	
Other	264 (7.8)	32 (7.9)	162 (8.0)	70 (7.3)	
None	38 (1.1)	7 (1.7)	24 (1.2)	7 (0.7)	
NYHA functional class					
II	421 (12.4)	33 (8.2)	253 (12.5)	135 (14.0)	<.001
III	1491 (44.0)	150 (37.1)	918 (45.4)	423 (44.0)	
IV	1474 (43.5)	221 (54.7)	850 (42.1)	403 (41.9)	
Comorbidities					
Coronary artery disease	1967 (58.1)	208 (51.5)	1194 (59.1)	565 (58.8)	.058
Valvular heart disease	590 (17.4)	74 (18.3)	352 (17.4)	164 (17.1)	.60
Hypertension	1920 (56.7)	181 (44.8)	1160 (57.4)	579 (60.2)	<.001
Atrial fibrillation	1301 (38.4)	150 (37.1)	782 (38.7)	369 (38.4)	.77
CRT implantation	36 (1.1)	5 (1.2)	24 (1.2)	7 (0.7)	.28
Pacemaker implantation	165 (4.9)	16 (4.0)	107 (5.3)	42 (4.4)	.88
ICD implantation	20 (0.6)	2 (0.5)	11 (0.5)	7 (0.7)	.53
Nonischemic cardiomyopathy	832 (24.6)	120 (29.7)	478 (23.7)	234 (24.3)	.14
Diabetes	1096 (32.4)	119 (29.5)	673 (33.3)	304 (31.6)	.78
Stroke	714 (21.1)	70 (17.3)	447 (22.1)	197 (20.5)	.51
COPD	666 (19.7)	85 (21.0)	393 (19.4)	188 (19.6)	.64
Reduced kidney function	1195 (35.3)	137 (33.9)	712 (35.2)	346 (36.0)	.47
Peripheral artery disease	379 (11.2)	34 (8.4)	223 (11.0)	122 (12.7)	.02
Anemia	773 (22.8)	87 (21.5)	478 (23.6)	208 (21.6)	.68
Cancer	132 (3.9)	7 (1.7)	82 (4.1)	43 (4.5)	.04
Laboratory testing and imaging					
Serum sodium level, median (IQR), mmol/L	140 (137-142)	139 (137-142)	140 (137-142)	140 (138-142)	.01
NT-proBNP level, median (IQR), pg/mL	2087 (791-4758)	2617 (1220-6371)	2184 (795-5105)	1673 (682-3855)	<.001
LVEF, %					
<40	1411 (41.7)	185 (45.8)	833 (41.2)	393 (40.9)	.47
≥40 to <50	818 (24.2)	103 (25.5)	481 (23.8)	234 (24.3)	
≥50	1157 (34.2)	116 (28.7)	707 (35.0)	334 (34.8)	
Clinical features at discharge					
Heart rate, median (IQR), beats/min	72 (67-80)	72 (68-80)	72 (67-80)	72 (66-79)	.057
SBP, median (IQR), mm Hg	120 (110-130)	120 (105-130)	120 (110-130)	120 (110-130)	<.001
Discharge medication					
ACEI or ARB	1720 (50.8)	190 (47.0)	995 (49.2)	535 (55.7)	<.001
β-Blocker	2000 (59.1)	223 (55.2)	1183 (58.5)	594 (61.8)	.02
Aldosterone antagonist	2176 (64.3)	269 (66.6)	1297 (64.2)	610 (63.5)	.32
Diuretics	2343 (69.2)	285 (70.5)	1407 (69.6)	651 (67.7)	.24

Abbreviations: ACEI, angiotensin-converting enzyme inhibitor; ARB, angiotensin receptor blocker; COPD, chronic obstructive pulmonary disease; CRT, cardiac resynchronization therapy; ICD, implantable cardioverter defibrillator; LVEF, left ventricular ejection fraction; NCMS, New Rural Cooperative Medical Scheme; NT-proBNP, N-terminal pro-B-type natriuretic peptide; NYHA, New York Heart Association; PEB, perceived economic burden; RMB, renminbi; SBP, systolic blood pressure; UEBMI, Urban Employee-Based Medical Insurance; URBMI, Urban Resident-Based Medical Insurance.

SI conversion factor: To convert serum sodium to mEq/L, multiply by 1.0.

^a^ Data are presented as the number (percentage) of patients unless otherwise indicated.

^b^ Based on the 2016 exchange rate.

### Associations Between PEB and Clinical Outcomes

**Figure 1.**
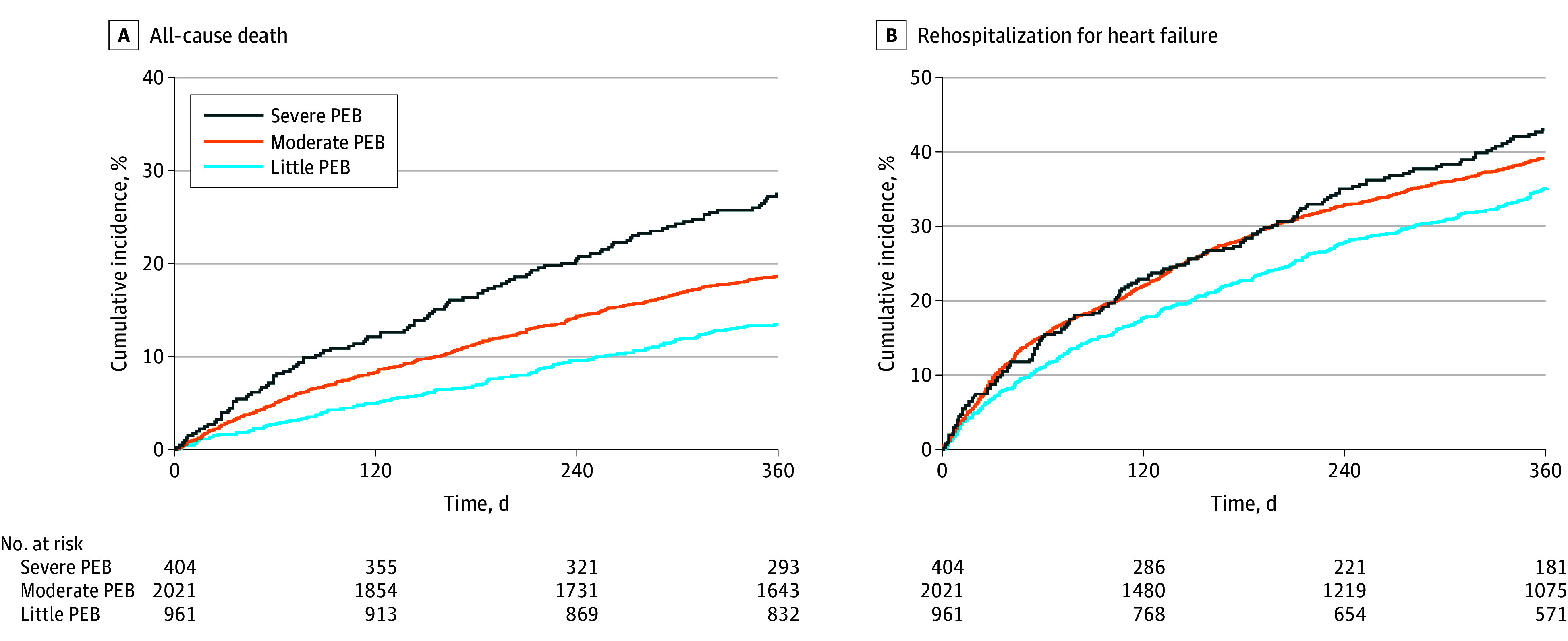
Kaplan-Meier Plots for Death and Rehospitalization for Heart Failure by Perceived Economic Burden (PEB) Little PEB was the reference category. A, For severe PEB, *P* < .001; for moderate PEB, *P* = .005. B, For severe PEB, *P* = .006; for moderate PEB, *P* = .04.

**Figure 2.**
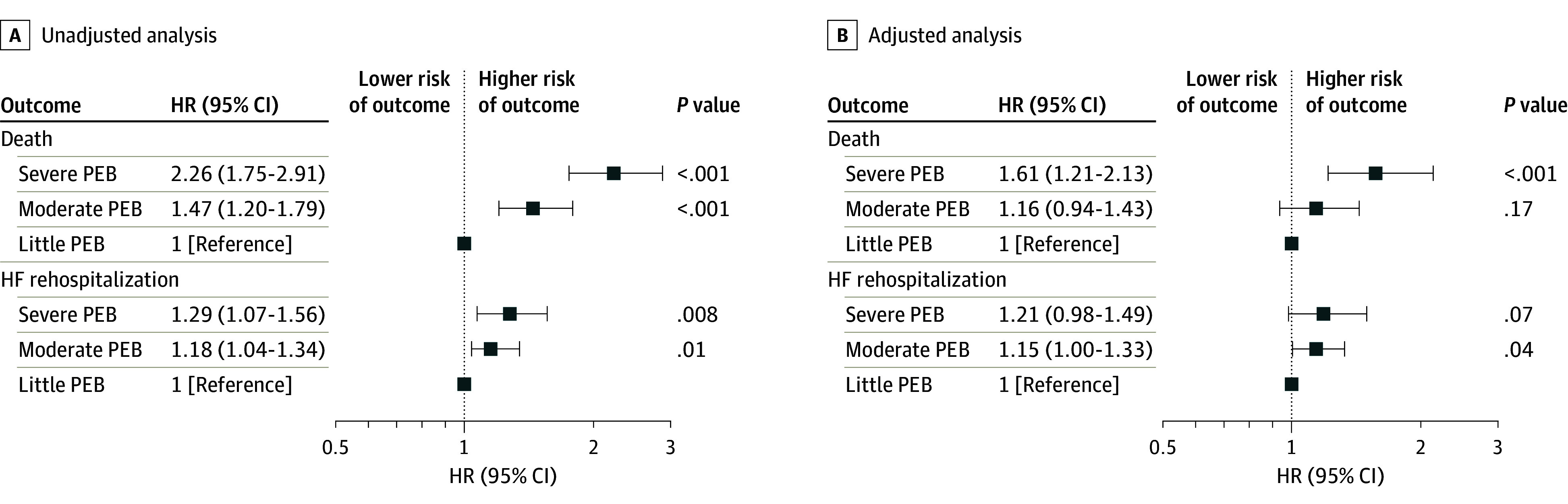
Risk of 1-Year Death and Rehospitalization for Heart Failure Among Patients With Severe or Moderate Perceived Economic Burden (PEB) Compared With Those With Little PEB HR indicates hazard ratio.

During 1-year follow-up, all-cause death occurred in 112 (27.7%), 383 (18.9%), and 129 (13.4%) patients with severe, moderate, and little PEB, respectively, and HF rehospitalization occurred in 159 (39.4%), 756 (37.4%), and 329 (34.2%), respectively (Figure 1). After adjustment for potential confounders, risk of 1-year mortality was significantly increased among patients with severe PEB (HR, 1.61; 95% CI, 1.21-2.13; *P* < .001) but not among patients with moderate PEB (HR, 1.16; 95% CI, 0.94-1.43; *P* = .17) compared with those with little PEB. Risk of 1-year HF rehospitalization was not increased in patients with severe PEB (HR, 1.21; 95% CI, 0.98-1.49; *P* = .07) but was increased in patients with moderate PEB (HR, 1.15; 95% CI, 1.00-1.33; *P* = .04) compared with those with little PEB (Figure 2). There were no interactions across subgroups except in the risk of HF rehospitalization in annual family income subgroups (eFigures 1 and 2 in Supplement 1).

After adjusting for baseline KCCQ-12 scores, severe PEB was still associated with increased risk of 1-year mortality (HR, 1.46; 95% CI, 1.10-1.94; *P* = .008) compared with little PEB (eTable 2 in Supplement 1). There were no interactions between KCCQ-12 scores, PEB, and clinical outcomes. In sensitivity analysis, the association between PEB and clinical outcomes in patients with new-onset HF were consistent with those in patients with ADHF (eTable 4 and eFigures 3 and 4 in Supplement 1).

### Associations Between PEB and Health Status

**Figure 3.**
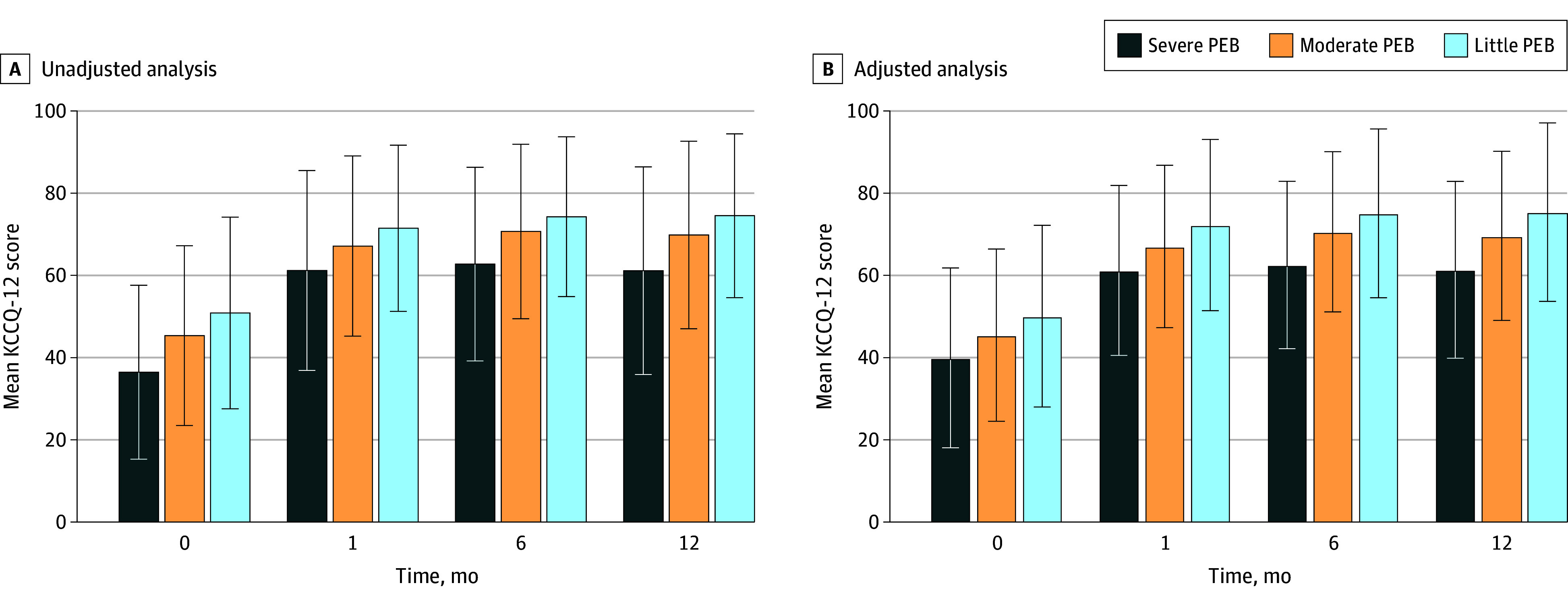
Unadjusted and Adjusted Analyses of 12-Item Kansas City Cardiomyopathy Questionnaire (KCCQ-12) Scores by Perceived Economic Burden (PEB) Scores range from 0 to 100; a higher score reflects possibly better health-related quality of life. Error bars represent SDs. Trend for all differences was significant at *P* < .001.

**Figure 4.**
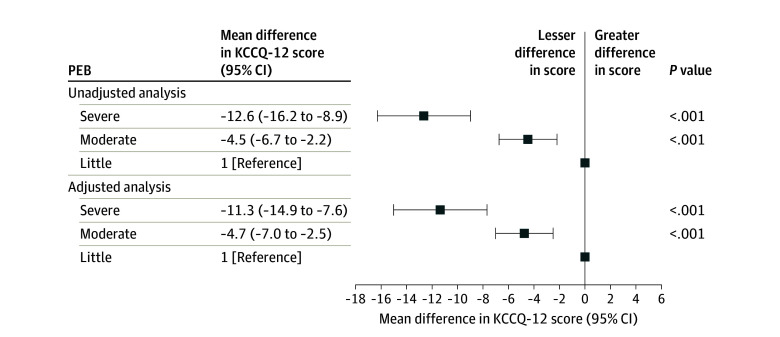
Difference in 1-Year 12-Item Kansas City Cardiomyopathy Questionnaire (KCCQ-12) Score in Patients With Severe or Moderate Perceived Economic Burden (PEB) Compared With Those With Little PEB Scores range from 0 to 100; a higher score reflects possibly better health-related quality of life.

There were 1633 patients included in the analysis of KCCQ-12 scores, accounting for 59.1% of the 2762 patients who were alive at 1-year follow-up. The 1129 patients (40.9%) excluded from this analysis were generally similar to those included, but they were somewhat older, had a higher LVEF, and had lower educational level and baseline KCCQ-12 scores (eTable 3 in Supplement 1). The unadjusted and adjusted mean (SD) KCCQ-12 scores from baseline to the 12-month follow-up visit are presented by PEB group in Figure 3. The mean KCCQ-12 score persistently improved from baseline to 6 months and remained stable at 12 months in all PEB groups. The mean KCCQ-12 scores were lowest in patients with severe PEB and highest in patients with little PEB burden at baseline and each visit. After adjustment, the trends in health status were consistent across PEB groups. The mean (SD) adjusted KCCQ-12 score was lowest in patients with severe PEB and highest in patients with little PEB at baseline (40.0 [1.7] and 50.2 [1.0] points, respectively; *P* < .001) and at each visit (eg, 12 months: 61.5 [1.6] and 75.5 [0.9] points respectively; *P* < .001). In the model adjusted for baseline KCCQ-12 score, patients reporting severe and moderate PEB had clinically significant lower 1-year KCCQ-12 scores (severe PEB: mean difference, −11.3 points; 95% CI, −14.9 to −7.6 points; *P* < .001; moderate PEB: mean difference, −4.7 points; 95% CI, −7.0 to −2.5 points; *P* < .001) compared with patients with little PEB (Figure 4).

## Discussion

In this large national cohort study in China, we demonstrated that greater PEB was associated with a higher risk of 1-year all-cause mortality and worse health status but not with HF rehospitalization. In this cohort, 11.9% of patients hospitalized for ADHF perceived severe economic burden and 59.6% of patients perceived moderate economic burden. Compared with patients with little PEB, those with severe PEB had a higher risk of mortality and a clinically significant lower 1-year KCCQ-12 score within 1 year of discharge. These findings indicate PEB as a potential factor to be considered for future intervention.

We extended earlier literature that found that PEB was associated with adverse clinical outcomes. A prior study found that socioeconomic status, including low income, unemployment status, low educational level, and unpartnered status, was associated with a higher risk of death in patients with HF.^24^ We demonstrated that the risk of mortality was higher among patients with severe PEB compared with their counterparts with little PEB after adjusting for all socioeconomic status factors assessed in our study. These results were consistent among specific population subgroups. Moreover, previous studies reported that patients with HF who perceived difficulty in affording their medical care had lower perceived health status.^10,11,18^ We extended prior findings by using a large national cohort. We demonstrated that PEB was associated with impaired health status, and the association was consistent during 1-year follow-up. Taken together, PEB was independently associated with ADHF outcomes.

The underlying mechanism of the association between PEB and adverse clinical outcomes was unknown. Previous studies reported that difficulties in affording health care expenditures was associated with mental health problems,^25^ medication nonadherence,^26^ delaying or forgoing medical care,^3^ and food insecurity,^4^ all of which may jeopardize longevity. Of note, more severe PEB was not associated with risk of HF rehospitalization after adjustment. This finding also supports the possibility that patients with severe PEB forgo timely hospitalization due to concern about medical costs. In addition, we found that severe PEB was associated with clinically significant deterioration in health status independent of baseline KCCQ-12 score, socioeconomic status, and other clinical characteristics. In a prior study, individuals who reported an inability to pay medical bills had more than 3-fold higher odds of subjective distress from health care finances.^4^ These health-related stressors may contribute to the damaged health status associated with PEB.

Heart failure is a prevalent, deadly chronic condition that requires recurrent examination, indefinite medication use, and even advanced devices. A scenario in which a patient is facing the threat of death with so many available treatment options may pressure physicians to prescribe these options while ignoring their cost. However, our results indicate that these costs might also be toxic by increasing the PEB, which may even completely neutralize the possible effects of these costly interventions. In clinical practice, 1 simple question probing a patient’s PEB may directly help clinical practitioners quickly identify patients who may experience economic burden. For patients with severe PEB, some further consideration of balancing the cost and benefits when selecting examinations and treatments may not only help relieve their burden but also be associated with improved prognosis.

Notably, we found that patients with medical insurance coverage by NCMS were more likely to perceive severe economic burden and those with UEBMI were more likely to perceive little economic burden. Although China has universal basic medical insurance coverage,^8,27^ a previous survey^8^ found that out-of-pocket costs were still high, which possibly resulted in financial difficulties. Moreover, insurance policies vary across regions (eg, rural and urban, east and west), populations (employed, unemployed), and occasions (inpatient, outpatient) in China, which contributes to payment disparities. The benefit packages available to urban populations were better than those available to rural populations.^28,29^ Based on data from the National Health Service Survey in 2014, the proportion of out-of-pocket payment for in-patient care was 32.5% for urban employees and 44.4% for rural residents.^30^ Outpatient costs can be largely reimbursed in URBMI^31^ but with small reimbursements in NCMS or URBMI.^32^ To alleviate PEB of medical care, policy interventions need to focus on financially vulnerable populations who are less protected by medical insurance.

The Chinese government has made great efforts to alleviate expenses in health care in the past decade. Creating equitable and affordable health care has been an established goal in many countries and was also raised as a principle in the Healthy China 2030 Initiative.^33^ China witnessed remarkable development in primary health care, reform in the health insurance system, and control of health care prices.^34,35^ For example, HF has been added to the list of noncommunicable diseases covered by outpatient insurance in many regions since 2020,^36^ which can substantially reduce outpatient expenses. More research is needed to address whether these steps mitigate perceived economic difficulties in affording health care. Additionally, further study is needed to determine whether reducing PEB can prolong longevity and improve patients’ health status.

### Limitations

The findings of our study should be interpreted in light of several limitations. First, the construct used to define PEB was not validated by objective measures of out-of-pocket costs for medical expenditures. However, similar items used to create the domain have been widely used in prior studies,^10,11,18,37^ and the severity of PEB in this study was shown to correlate well with the likelihood of borrowing money from others to pay for medical expenses. Second, there might be potential unmeasured confounders that influenced the reliability of our conclusions, and adjustments were insufficient in this regard. However, these are the inherent limitations of an observational study. Third, 40.9% of patients were not included in the analysis of KCCQ-12 scores because they did not complete all follow-up KCCQ-12 assessments. Fourth, external validity of the findings is limited since financial, social, and insurance situations may be different between China and other countries.

## Conclusions

In this cohort study of patients with ADHF in China, we found that more perceived financial burden from health care costs was associated with higher mortality and poorer quality of life but not with HF rehospitalization. National efforts should be expanded to identify pragmatic approaches not only to alleviate the PEB in health care but also to assess its potential consequences for saving lives and improving health status.

## Notes

Supplement 1. **eTable 1.** Perceived Economic Burden and Reported Borrowing Money, Avoiding Healthcare Due to Costs and Medical Expense **eTable 2.** Risk of 1-Year Death and HF Hospitalization for Severe and Moderate PEB Compared With Little PEB After Adjusting for Baseline KCCQ-12 **eTable 3.** Baseline Characteristics Between Patients Included and Excluded in the Analyses of KCCQ-12 **eTable 4.** Baseline Demographic and Socioeconomic Characteristics of Patients With New Onset Heart Failure by Perceived Economic Burden **eFigure 1.** Risk of 1-Year Death for Severe and Moderate PEB Compared With Little PEB in Various Subgroups **eFigure 2.** Risk of 1-Year HF Rehospitalization for Severe and Moderate PEB Compared With Little PEB in Various Subgroups **eFigure 3.** Kaplan-Meier Plots for Death and HF Rehospitalization by Perceived Economic Burden Among Patients With New Onset Heart Failure **eFigure 4.** Risk of 1-Year Death and HF Rehospitalization for Severe and Moderate Perceived Economic Burden Compared With Little Burden Among Patients With New Onset Heart Failure

Supplement 2.
Data Sharing Statement
