# Supplementary material for: Perceived Economic Burden, Mortality, and Health Status in Patients With Heart Failure
Source: JAMA Netw Open. 2024 Mar 21;7(3):e241420. doi: 10.1001/jamanetworkopen.2024.1420 (PMC10958235; doi:10.1001/jamanetworkopen.2024.1420)
Supplement: Supplement 1. — eTable 1. Perceived Economic Burden and Reported Borrowing Money, Avoiding Healthcare Due to Costs and Medical Expense eTable 2. Risk of 1-Year Death and HF Hospitalization for Severe and Moderate PEB Compared With Little PEB After Adjusting for Baseline KCCQ-12 eTable 3. Baseline Characteristics Between Patients Included and Excluded in the Analyses of KCCQ-12 eTable 4. Baseline Demographic and Socioeconomic Characteristics of Patients With New Onset Heart Failure by Perceived Economic Burden eFigure 1. Risk of 1-Year Death for Severe and Moderate PEB Compared With Little PEB in Various Subgroups eFigure 2. Risk of 1-Year HF Rehospitalization for Severe and Moderate PEB Compared With Little PEB in Various Subgroups eFigure 3. Kaplan-Meier Plots for Death and HF Rehospitalization by Perceived Economic Burden Among Patients With New Onset Heart Failure eFigure 4. Risk of 1-Year Death and HF Rehospitalization for Severe and Moderate Perceived Economic Burden Compared With Little Burden Among Patients With New Onset Heart Failure [file jamanetwopen-e241420-s001.pdf]

Supplementary Online Content

Yu Y, Liu J, Zhang L, et al. Perceived economic burden, mortality, and health status in patients with heart failure. *JAMA Netw Open*. 2024;7(3):e241420. doi:10.1001/jamanetworkopen.2024.1420

eTable 1. Perceived Economic Burden and Reported Borrowing Money, Avoiding Healthcare Due to Costs and Medical Expense

eTable 2. Risk of 1-Year Death and HF Hospitalization for Severe and Moderate PEB Compared With Little PEB After Adjusting for Baseline KCCQ-12

eTable 3. Baseline Characteristics Between Patients Included and Excluded in the Analyses of KCCQ-12

eTable 4. Baseline Demographic and Socioeconomic Characteristics of Patients With New Onset Heart Failure by Perceived Economic Burden

eFigure 1. Risk of 1-Year Death for Severe and Moderate PEB Compared With Little PEB in Various Subgroups

eFigure 2. Risk of 1-Year HF Rehospitalization for Severe and Moderate PEB Compared With Little PEB in Various Subgroups

eFigure 3. Kaplan-Meier Plots for Death and HF Rehospitalization by Perceived Economic Burden Among Patients With New Onset Heart Failure

eFigure 4. Risk of 1-Year Death and HF Rehospitalization for Severe and Moderate Perceived Economic Burden Compared With Little Burden Among Patients With New Onset Heart Failure

This supplementary material has been provided by the authors to give readers additional information about their work.

© 2024 Yu Y et al. *JAMA Network Open*.

eTable 1. Perceived economic burden and reported borrowing money, avoiding healthcare due to costs and medical expense.

	All (n=3386)	Severe (n=404)	Moderate (n=2021)	Little (n=961)	P trend
**In the past 12 month, have you ever borrowed money from others to pay for medical expense? n(%)**					<0.001
Yes	407 (12.0)	239 (59.2)	156 (7.7)	12 (1.2)	
No	2852 (84.2)	144 (35.6)	1773 (87.7)	935 (97.3)	
Unknown	127 (3.8)	21 (5.2)	92 (4.6)	14 (1.5)	
**In the past 12 month, have you ever avoided healthcare due to costs? n(%)**					<0.001
Yes	740 (21.8)	261 (64.6)	435 (21.5)	44 (4.6)	
No	2565 (75.8)	137 (33.9)	1524 (75.4)	904 (94.1)	
Unknown	81 (2.4)	6 (1.5)	62 (3.1)	13 (1.3)	
**Medical expense, median (IQR), RMB**					
Index hospitalization*	9555 (7051-13085)	9243 (6974-13045)	9686 (7115-13179)	9378 (6937-12749)	0.540
Annual out-of-pocket*	6067 (3500-11152)	14000 (6000-25000)	7000 (4000-12000)	4000 (2000-7144)	<0.001

IQR indicates interquartile range; RMB, renminbi.
*The missing rate of both medical expense of index hospitalization and annual out-of-pocket payment were 34.5%.

© 2024 Yu Y et al. *JAMA Network Open*.

eTable 2. Risk of 1-year death and HF hospitalization for severe and moderate PEB compared with little PEB after adjusting baseline KCCQ-12.

**Outcomes**	**HR (95% CI)**	**P value**	**P for interaction***
**Death**			
PEB			0.211
Severe	1.46 (1.10-1.94)	0.008	
Moderate	1.11 (0.89-1.37)	0.356	
KCCQ-12	0.99 (0.99-0.99)	<0.001	
**HF rehospitalization**			
PEB			0.367
Severe	1.16 (0.94-1.43)	0.171	
Moderate	1.13 (0.98-1.30)	0.083	
KCCQ-12	0.99 (0.99-1.00)	<0.001	

PEB indicates perceived economic burden; KCCQ-12, 12-item Kansas City Cardiomyopathy Questionnaire.

*P for interaction indicates the p value for interactions between KCCQ-12 and PEB.

© 2024 Yu Y et al. *JAMA Network Open*.

eTable 3. Baseline characteristics between patients included and excluded in the analyses of KCCQ-12.

	All (n=2773)	Included in the analyses of KCCQ-12		P
		Yes (n=1633)	No (n=1140)	
**Sociodemographic**				
Age, median (IQR), y	67 (57-75)	66 (56-74)	68 (58-76)	<0.001
Women, n(%)	1041 (37.5)	587 (36.0)	454 (39.8)	0.038
Current smoker, n(%)	474 (17.1)	315 (19.3)	159 (14.0)	<0.001
Married, n(%)	2267 (81.8)	1331 (81.5)	936 (82.1)	0.688
High school education or above, n(%)	810 (29.2)	512 (31.4)	298 (26.1)	0.003
Annual family income, n(%), RMB				0.009
<30000	942 (34.0)	529 (32.4)	413 (36.2)	
30000-70000	941 (33.9)	586 (35.9)	355 (31.1)	
>70000	387 (14.0)	240 (14.7)	147 (12.9)	
Unknown	503 (18.1)	278 (17.0)	225 (19.7)	
**NYHA functional class, n(%)**				0.771
II	386 (13.9)	221 (13.5)	165 (14.5)	
III	1258 (45.4)	746 (45.7)	512 (44.9)	
IV	1129 (40.7)	666 (40.8)	463 (40.6)	
**Comorbidities, n(%)**				
Coronary artery disease	1613 (58.2)	990 (60.6)	623 (54.6)	0.002
Valvular heart disease	469 (16.9)	266 (16.3)	203 (17.8)	0.294
Hypertension	1604 (57.8)	947 (58.0)	657 (57.6)	0.850
Atrial fibrillation	1062 (38.3)	616 (37.7)	446 (39.1)	0.455
Nonischemic cardiomyopathy	686 (24.7)	411 (25.2)	275 (24.1)	0.530
Diabetes mellitus	889 (32.1)	541 (33.1)	348 (30.5)	0.148
Stroke	576 (20.8)	353 (21.6)	223 (19.6)	0.189
COPD	513 (18.5)	289 (17.7)	224 (19.6)	0.193
Reduced renal function	911 (32.8)	499 (30.6)	412 (36.1)	0.002
Peripheral artery disease	315 (11.4)	179 (11.0)	136 (11.9)	0.429
Anemia	565 (20.4)	303 (18.6)	262 (23.0)	0.004
Cancer	95 (3.4)	68(4.2)	27 (2.4)	0.011
**laboratory testing and imaging**				

© 2024 Yu Y et al. *JAMA Network Open*.

Natrium, median (IQR), mmol/L	140 (138-142)	140 (138-142)	140 (137-142)	0.007
NT-proBNP, median (IQR), pg/mL	1801 (701-4011)	1744 (677-3960)	1897 (735-4078)	0.092
LVEF, n(%)				
<40%	1074 (38.7)	641 (39.2)	433 (38.0)	0.019
≥40%, <50%	602 (21.7)	381 (23.3)	221 (19.4)	
≥50%	949 (34.2)	531 (32.5)	418 (36.7)	
**Clinical features at discharge**				
Heart rate, median (IQR), bpm	72 (67-80)	72 (66-78)	72 (68-80)	<0.001
SBP, median (IQR), mmHg	120 (110-130)	120 (110-130)	120 (110-130)	0.254
**Discharge medication, n(%)**				
ACEI or ARB	1463 (52.8)	839 (51.4)	624 (54.7)	0.081
Beta blocker	1683 (60.7)	1008 (61.7)	675 (59.2)	0.182
Aldosterone antagonist	1772 (63.9)	1029 (63.0)	743 (65.2)	0.243
Diuretics	1898 (68.4)	1083 (66.3)	815 (71.5)	0.004
**Baseline KCCQ-12 score**, median (IQR)	44 (28-61)	46 (30-63)	42 (26-58)	<0.001

COPD indicates chronic obstructive pulmonary disease; IQR, interquartile range; LVEF, left ventricular ejection fraction; NYHA, New York Heart Association; SBP, systolic blood pressure; ACEI, angiotensin-converting enzyme inhibitor; ARB, angiotensin receptor blocker; KCCQ-12, Kansas City Cardiomyopathy Questionnaire.

© 2024 Yu Y et al. *JAMA Network Open*.

eTable 4. Baseline demographic and socioeconomic characteristics of patients with new onset heart failure by perceived economic burden.

	All (n=853)	Severe (n=63)	Moderate (n=445)	Little (n=345)	P trend
Age, median (IQR), y	64 (53-75)	61 (50-68)	67 (57-76)	62 (50-71)	0.004
Women, n(%)	308 (36.1)	26 (41.3)	189 (42.5)	93 (27.0)	<0.001
Current smoker, n(%)	241 (28.2)	17 (27.0)	102 (22.9)	122 (35.4)	0.002
Married, n(%)	690 (80.9)	47 (74.6)	350 (78.6)	293 (84.9)	0.010
High school education or above, n(%)	256 (30.0)	8 (12.7)	113 (25.4)	135 (39.1)	<0.001
Annual family income, n(%), RMB					0.952
<30000	278 (32.6)	41 (65.1)	165 (37.1)	72 (20.9)	
30000-70000	291 (34.1)	9 (14.3)	147 (33.0)	135 (39.1)	
>70000	107 (12.5)	0 (0)	38 (8.5)	69 (20.0)	
Unknown	177 (20.8)	13 (20.6)	95 (21.4)	69 (20.0)	
**NYHA functional class, n(%)**					0.690
II	144 (16.9)	7 (11.1)	82 (18.4)	55 (15.9)	
III	359 (42.1)	25 (39.7)	186 (41.8)	148 (42.9)	
IV	350 (41.0)	31 (49.2)	177 (39.8)	142 (41.2)	

© 2024 Yu Y et al. *JAMA Network Open*.

**Comorbidities, n(%)**					
Coronary artery disease	477 (55.9)	30 (47.6)	260 (58.4)	187 (54.2)	0.937
Valvular heart disease	102 (12.0)	9 (14.3)	56 (12.6)	37 (10.7)	0.321
Hypertension	520 (61.0)	39 (61.9)	270 (60.7)	211 (61.2)	0.992
Atrial fibrillation	238 (27.9)	15 (23.8)	121 (27.2)	102 (29.6)	0.296
Nonischemic cardiomyopathy	181 (21.2)	17 (27.0)	81 (18.2)	83 (24.1)	0.396
Diabetes mellitus	260 (30.5)	19 (30.2)	138 (31.0)	103 (29.9)	0.811
Stroke	155 (18.2)	11 (17.5)	90 (20.2)	54 (15.6)	0.228
COPD	133 (15.6)	13 (20.6)	77 (17.3)	43 (12.5)	0.030
Reduced renal function	238 (27.9)	17 (27.0)	134 (30.1)	87 (25.2)	0.276
Peripheral artery disease	106 (12.4)	7 (11.1)	55 (12.4)	44 (12.8)	0.738
Anemia	194 (22.7)	13 (20.6)	115 (25.8)	66 (19.1)	0.134
Cancer	37 (4.3)	3 (4.8)	25 (5.6)	9 (2.6)	0.085
**laboratory testing and imaging**					
Sodium, median (IQR), mmol/L	140 (138-142)	140 (137-142)	140 (138-142)	140 (138-142)	0.230
NT-proBNP, median (IQR), pg/mL	1710 (740-3801)	2728 (1182-6444)	1838 (734-4313)	1457 (699-3282)	0.020
LVEF, n(%)					0.130

© 2024 Yu Y et al. *JAMA Network Open*.

<40%	288 (33.8)	29 (46.0)	130 (29.2)	129 (37.4)	
≥40%, <50%	202 (23.7)	8 (12.7)	105 (23.6)	89 (25.8)	
≥50%	338 (39.6)	22 (34.9)	195 (43.8)	121 (35.1)	
**Clinical features at discharge**					
Heart rate, median (IQR), bpm	72 (68-80)	76 (70-81)	72 (68-80)	72 (67-80)	0.066
SBP, median (IQR), mmHg	122 (110-132)	122 (115-133)	121 (110-132)	122 (112-131)	0.672
**Discharge medication, n(%)**					
ACEI or ARB	461 (54.0)	36 (57.1)	223 (50.1)	202 (58.6)	0.124
Beta blocker	527 (61.8)	29 (46.0)	268 (60.2)	230 (66.7)	0.002
Aldosterone antagonist	531 (62.2)	43 (68.2)	266 (59.8)	222 (64.4)	0.688
Diuretics	585 (68.6)	46 (73.0)	299 (67.2)	240 (69.6)	0.942

COPD indicates chronic obstructive pulmonary disease; IQR, interquartile range; LVEF, left ventricular ejection fraction; NYHA, New York Heart Association; SBP, systolic blood pressure; ACEI, angiotensin-converting enzyme inhibitor; ARB, angiotensin receptor blocker.

© 2024 Yu Y et al. *JAMA Network Open*.

eFigure 1. Risk of 1-year death for severe and moderate PEB compared with little PEB in various subgroups.

Subgroup	No. of patients	Severe PEB	HR (95% CI)	Moderate PEB	HR (95% CI)	P for interaction
Age, y						0.931
<=65	1545		1.78 (1.15-2.76)		1.24 (0.86-1.78)	
>65	1841		1.51 (1.03-2.20)		1.09 (0.84-1.42)	
Gender						0.934
Men	2116		1.46 (1.02-2.09)		1.17 (0.90-1.51)	
Women	1270		2.08 (1.29-3.35)		1.21 (0.83-1.79)	
Annual family income (RMB)						0.687
<30000	1185		1.91 (1.21-3.02)		1.37 (0.91-2.08)	
30000-70000	1133		1.78 (1.03-3.07)		1.25 (0.87-1.81)	
>70000	449		0.45 (0.05-3.94)		0.82 (0.46-1.47)	
Unknown	619		1.58 (0.76-3.30)		1.25 (0.75-2.08)	
Medical insurance						0.986
UEBMI	1445		1.67 (0.98-2.86)		1.19 (0.88-1.61)	
URBMI	619		2.11 (1.06-4.17)		1.15 (0.68-1.95)	
NCMS	1020		1.65 (0.99-2.74)		1.26 (0.80-1.98)	
Other	264		1.11 (0.38-3.24)		0.82 (0.35-1.93)	
Reference, little PEB						

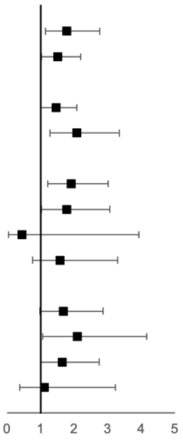

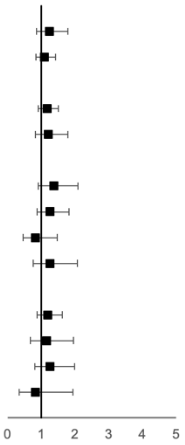

PEB indicates perceived economic burden; UEBMI, Urban Employee-based Basic Medical Insurance; URBMI, Urban Resident-based Basic Medical Insurance; NCMS, New Rural Cooperative Medical scheme.

© 2024 Yu Y et al. *JAMA Network Open*.

eFigure 2. Risk of 1-year HF rehospitalization for severe and moderate PEB compared with little PEB in various subgroups.

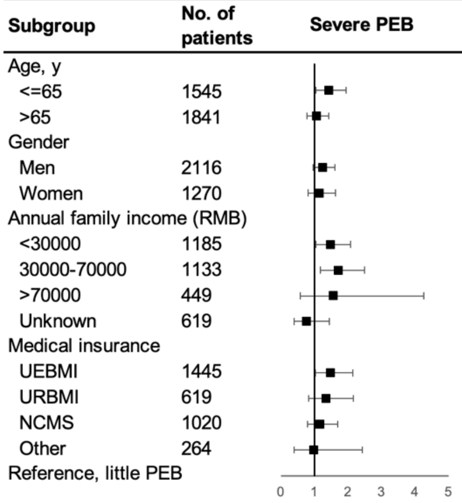

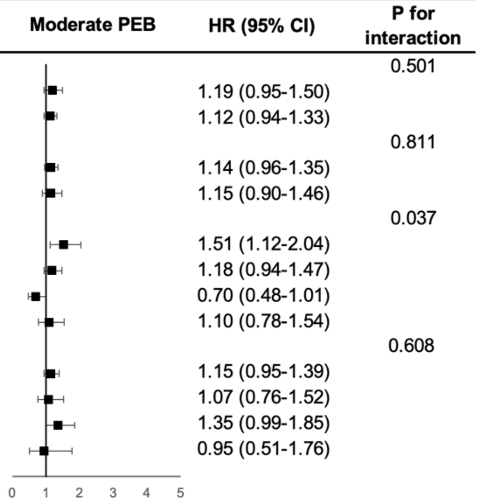

Subgroup	No. of patients	Severe PEB HR (95% CI)	Moderate PEB HR (95% CI)	P for interaction
Age, y				0.501
<=65	1545	1.43 (1.06-1.94)	1.19 (0.95-1.50)	
>65	1841	1.06 (0.79-1.43)	1.12 (0.94-1.33)	
Gender				0.811
Men	2116	1.25 (0.96-1.62)	1.14 (0.96-1.35)	
Women	1270	1.16 (0.82-1.64)	1.15 (0.90-1.46)	
Annual family income (RMB)				0.037
<30000	1185	1.48 (1.05-2.09)	1.51 (1.12-2.04)	
30000-70000	1133	1.71 (1.17-2.49)	1.18 (0.94-1.47)	
>70000	449	1.57 (0.58-4.26)	0.70 (0.48-1.01)	
Unknown	619	0.76 (0.40-1.45)	1.10 (0.78-1.54)	
Medical insurance				0.608
UEBMI	1445	1.49 (1.04-2.15)	1.15 (0.95-1.39)	
URBMI	619	1.34 (0.83-2.16)	1.07 (0.76-1.52)	
NCMS	1020	1.16 (0.80-1.70)	1.35 (0.99-1.85)	
Other	264	0.98 (0.40-2.43)	0.95 (0.51-1.76)	
Reference, little PEB				

PEB indicates perceived economic burden; UEBMI, Urban Employee-based Basic Medical Insurance; URBMI, Urban Resident-based Basic Medical Insurance; NCMS, New Rural Cooperative Medical scheme.

© 2024 Yu Y et al. *JAMA Network Open*.

eFigure 3. Kaplan-Meier plots for death and HF rehospitalization by perceived economic burden among patients with new onset heart failure.

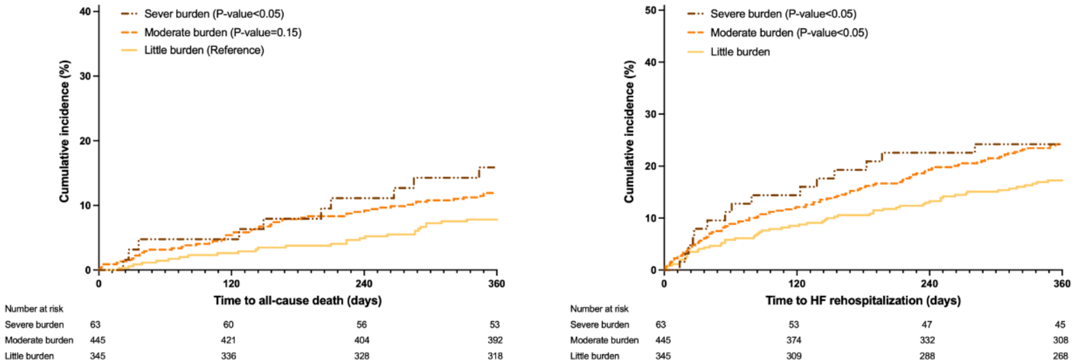

© 2024 Yu Y et al. *JAMA Network Open*.

eFigure 4. Risk of 1-year death and HF rehospitalization for severe and moderate perceived economic burden compared with little burden among patients with new onset heart failure.

Outcomes	Unadjusted	HR (95% CI)	P value	Adjusted	HR (95% CI)	P value
**Death**						
Severe		1.98 (0.96-4.06)	0.063		1.26 (0.56-2.84)	0.568
Moderate		1.49 (0.95-2.34)	0.084		1.02 (0.62-1.67)	0.939
Little		Reference			Reference	
**HF rehospitalization**						
Severe		1.51 (0.86-2.67)	0.152		1.35 (0.72-2.54)	0.353
Moderate		1.49 (1.08-2.06)	0.014		1.24 (0.86-1.78)	0.240
Little		Reference			Reference	

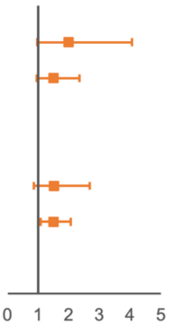

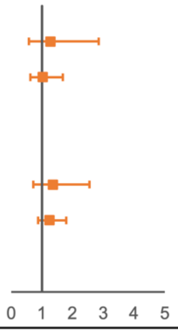

© 2024 Yu Y et al. *JAMA Network Open*.
