# Supplementary material for: Perceived Economic Burden, Mortality, and Health Status in Patients With Heart Failure
Source: JAMA Netw Open. 2024 Mar 21;7(3):e241420. doi: 10.1001/jamanetworkopen.2024.1420 (PMC10958235; doi:10.1001/jamanetworkopen.2024.1420)
Supplement: Supplement 2. — Data Sharing Statement [file jamanetwopen-e241420-s002.pdf]

Data Sharing Statement

Yu. Perceived Economic Burden, Mortality, and Health Status in Patients With Heart Failure. *JAMA Netw Open*. Published March 21, 2024. doi:10.1001/jamanetworkopen.2024.1420

Data
Data available: No
